# Identification of fibrillin 1 gene mutations in patients with bicuspid aortic valve (BAV) without Marfan syndrome

**DOI:** 10.1186/1471-2350-15-23

**Published:** 2014-02-24

**Authors:** Guglielmina Pepe, Stefano Nistri, Betti Giusti, Elena Sticchi, Monica Attanasio, Cristina Porciani, Rosanna Abbate, Robert O Bonow, Magdi Yacoub, Gian Franco Gensini

**Affiliations:** 1 Department of Experimental and Clinical Medicine, Section of Critical Medical Care and Medical Specialities; DENOTHE Center, University of Florence, Largo Brambilla 3, 50134 Florence, Italy; 2 Department of Heart and Vessels, Regional Marfan Syndrome and Related Disorders Center, Careggi Hospital, Florence, Italy; 3 Cardiology Service, CMSR Veneto Medica, AltavillaVicentina, Italy; 4 Department of Medicine, Northwestern University, Chicago, IL, USA; 5 Heart Science Centre, Imperial College, London, UK; 6 S. Maria agli Ulivi Center, Fondazione Don Carlo Gnocchi, Onlus, IRCCS, Florence, Italy

**Keywords:** Bicuspid aortic valve, Aortic disease, Aneurysm, Marfan syndrome, Fibrillin-1

## Abstract

**Background:**

Bicuspid aortic valve (BAV) is the most frequent congenital heart disease with frequent involvement in thoracic aortic dilatation, aneurysm and dissection. Although BAV and Marfan syndrome (MFS) share some clinical features, and some MFS patients with BAV display mutations in *FBN1*, the gene encoding fibrillin-1, the genetic background of isolated BAV is poorly defined.

**Methods:**

Ten consecutive BAV patients [8 men, age range 24–42 years] without MFS were clinically characterized. BAV phenotype and function, together with evaluation of aortic morphology, were comprehensively assessed by Doppler echocardiography. Direct sequencing of each *FBN1* exon with flanking intron sequences was performed on eight patients.

**Results:**

We detected three *FBN1* mutations in two patients (aged 24 and 25 years) displaying aortic root aneurysm ≥50 mm and moderate aortic regurgitation. In particular, one patient had two mutations (p.Arg2726Trp and p.Arg636Gly) one of which has been previously associated with variable Marfanoid phenotypes. The other patient showed a pArg529Gln substitution reported to be associated with an incomplete MFS phenotype.

**Conclusions:**

The present findings enlarge the clinical spectrum of isolated BAV to include patients with BAV without MFS who have involvement of *FBN1* gene. These results underscore the importance of accurate phenotyping of BAV aortopathy and of clinical characterization of BAV patients, including investigation of systemic connective tissue manifestations and genetic testing.

## Background

Bicuspid aortic valve (BAV) is the most common congenital heart disease [1,2]. BAV and Marfan syndrome (MFS) share some clinical features such as the increased prevalence of thoracic aortic aneurysm (TAA) and dissection (TAD), as well as overlapping histopathological features [3]. MFS is associated with mutations in the fibrillin 1 (*FBN1*) gene in more than 90% of patients, and less commonly in the transforming growth factor beta receptor 2 and 1 (*TGFBR2*, and *TGFBR1*) genes [4]. While the genetic background of MFS is well described at present, that of BAV is poorly defined.

BAV has been associated with *NOTCH1* gene mutations in a few cases [5], and with actin alpha 2 smooth muscle aorta (*ACTA2*) gene mutations (12%) in a subgroup of patients with TAA, livedo reticularis on the upper and lower limbs, and iris flocculi [6]. Other genes are suspected to be associated, and more chromosomal loci associated to BAV have been reported [7,8].

We have recently demonstrated a 4-fold increase in the prevalence of BAV in a large cohort of unrelated MFS patients (12 BAV in 257 MFS, 4.7%) with respect to the general population screened by echocardiography including primary school students in Italy (0.5%), detecting *FBN1* mutations in 2 out of 3 subjects who consented to undergo DNA mutation analysis [9]. These findings are consistent with data showing decreased *FBN1* mRNA or protein content in a subgroup of BAV patients [10], which suggest that *FBN1* may be one of the genes associated with BAV.

To date, however, the demonstration of *FBN1* mutations in patients with BAV is lacking. Thus, we screened for *FBN1* mutations in the selected patients with BAV and thoracic aortic dilation not fulfilling the clinical criteria for MFS.

## Methods

### Ethical statements

The local Ethical Committee of the Medicine Faculty of Florence approved the study protocols and participants provided their written informed consent to participate in this study.

### Subjects

Among the 432 patients with thoracic aortic dilatation, aneurysm, or dissection consecutively referred to the Center for Marfan Syndrome (Careggi Hospital, Florence, Italy) between 2001 and 2011, 22 were affected by BAV and aortic enlargement (aortic diameter ≥40 mm), of whom 12 had also MFS [9]. In the other ten patients, the diagnosis of MFS was excluded according to both the old and the revised Ghent criteria [4,11], and these patients constitute the subject of the present study. We also screened a control cohort of 200 unrelated individuals recruited from the same geographical area (160 male; mean age33.2 ± 8.5 years) for the presence of the genetic variants identified in the BAV patients with aortic enlargement. The controls were also evaluated for the presence and familial history of connective tissue disorders, BAV and aortic dilatation/dissection. The presence of BAV and or aortic dilatation/dissection was excluded in controls by echocardiography.

### Echocardiographic methods

All echocardiographic measurements had been made by a senior cardiologist (C.P.). BAV was diagnosed when only two cups were unequivocally identified in systole and diastole in the short axis view with a clear “fishmouth” appearance during systole as previously described [1,12]. Aortic dimensions were assessed at end-diastole in the parasternal long-axis view at four levels by the leading edge method [1,12,13] and Z-scores were calculated according to age-adjusted nomograms [13]. For patients who had undergone aortic surgery, still-frame photographs and/or available videos were also reviewed to verify and confirm the diagnosis of BAV. Patients for whom aortic valve morphology was indeterminate were considered as having tricuspid aortic valves. Aortic or mitral regurgitation were graded by multiple criteria combining color Doppler and continuous wave Doppler signals, and aortic valve stenosis was graded by peak aortic valve velocity [1].

### DNA extraction, polymerase chain reaction and direct sequencing

Genomic DNA was extracted from peripheral venous blood using FlexiGene Kit (Qiagen, Germany). The 65 exons of *FBN1* gene with the intronic flanking regions were amplified by polymerase chain reaction (PCR) [14]. PCR products were directly sequenced [14].

DNA samples from 200 healthy individuals were screened to determine whether the mutations identified in this study were present in a control population by direct sequencing of the exons in which the three mutations were identified. Prediction of the effect of mutations was performed by Polyphen-2 (http://genetics.bwh.harvard.edu/pph2/), Sorting Tolerant From Intolerant (SIFT, http://sift.jcvi.org) and MuPro (http://www.igb.uci.edu/~baldig/mutation.html) algorithms.

## Results

All patients [8 men and 2 women, age range 24–42 years] were Italian. Two of them (P8 and P10) (Table 1) had MASS syndrome (an acronym for myopia, mitral valve prolapse, aortic dilatation, skeleton features, skin features); most displayed systemic features such as pectus excavatum, scoliosis, pes planus, cutaneous striae, mitral valve prolapse and myopia (Table 1). All had fusion of the right and left coronary aortic valve cusps, and 8 of 10 patients had maximum aortic enlargement at the level of the aortic root and not at the ascending aorta.

**Table 1:** Clinical and molecular characteristics of the 10 studied patients

**Patients ID**	**Age at diagnosis/referred to our center (years)**	**Sex**	**Diagnostic criteria**								
			**BAV morphology**	**MAS: diameter (mm)/site**	**BAV hemodynamics**	**MVP**	**Eye EL**	**Systemic features**	**Systemic features score**	**Family history**	**FBN1 mutations**
P1	15/24	M	RL	50/AoR	AR moderate	-	-	-	0	TAA	Arg529Gln
P2	19/25	M	RL	57/AoR	AR moderate	+	-	MVP, My	2	-	Arg636Gly
											Arg2726Trp
P3	35/40	M	RL	45/AoR	-	-	-	My, PE, Sc, Th+	4	-	-
P4	27/40	F	RL	41/AoR	AR mild	+	-	MVP, PP, CS	3	TAA/AAA	-
P5	17/24	M	RL	47/AscA	-	-	-	Sc, PP, CS	3	-	-
P6	40/40	M	RL	44/AoR	-	-	-	PE, CS, facies	3	-	-
P7	42/42	M	RL	Prothesic tube	-	-	-	CS	1	-	na
P8	31/31	M	RL	42/AscA	-	+	-	MVP, PE, PC, PP, CS	6	BAV	na
P9	24/24	M	RL	40/AoR	-	-	-	PE, Ky, CS, My	4	TAA	-
P10	31/31	F	RL	48/AoR	AR mild	+	-	MVP, CS, DE, PP, Sc	5	-	-

ID = identification number; BAV = bicuspid aortic valve; MAS = maximal aortic size; M = male; F = female; MVP = mitral valve prolapse; RL = fusion of right and left coronary leaflets; EL = ectopia lentis; AoR = aortic root; AR = aortic regurgitation; AscA = Ascending aorta; Systemic features are reported and quoted according to new Ghent criteria (Loeys’ et al. 2010); CS = cutaneous Striae; EL = ectopia lentis; HD = hindfoot deformities; My = myopia; PE = pectus excavatum, ; PP = pes planus; Sc = scoliosis;Ky = kyphosis; Th + =positive thumb sign; + = present; - = absent; na = not analyzed.

### *FBN1* gene mutation analysis

Mutations analysis was performed on 8 of the 10 patients as P7 and P8 did not give consent to undergo mutation screening analysis. *FBN1* mutations were detected in two patients (P1 and P2).

**Figure 1:**
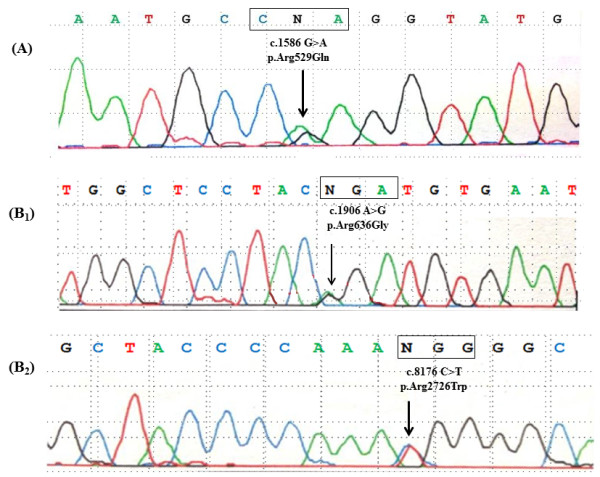
**Identification of *FBN1 *mutations in BAV patients. A**: Sequence chromatogram showing c.1586 G > A (p. Arg529Gln) mutation, identified in P1 patient. **B1/B2**: Sequence chromatograms showing c.1906 A > G (p. Arg636Gly) and c.8176C > T (p. Arg2726Trp) mutations, identified in P2 patients. Arrows indicate the locations of the point mutations.

P1 had a c.1586G > A, p.Arg529Gln mutation that represents a basic to polar neutral charge change in exon 12 (cbEGF-like 03 domain) (Figure 1). In P2 a double mutation was detected: a c.1906A > G mutation (p.Arg636Gly basic to apolar substitution) and a c.8176C > T mutation (p.Arg2726Trp causing a basic to apolar change); the first located in exon 15 (cbEGF-like 06 domain), the second in exon 64 (COOH unique region) (Figure 1). The three mutations were not present in 400 alleles among Italian controls. No mutations were detected in the other 6 patients.

## Discussion

To the best of our knowledge, this is the first study reporting pathogenetic fibrillin 1 mutations in patients with BAV and aortic dilatation/aneurysm in whom MFS and other more severe type 1 fibrillinopathies were clinically excluded according to the updated Ghent criteria [4].

The mutations detected in two unrelated patients are all arginine substitutions. The Arg529Gln mutation detected in P1 is reported at the UMD-FBN1 database (http://www.umd.be/FBN1/4DACTION/WV/2451) in a male proband of France geographic origin with an incomplete MFS phenotype. Unfortunately, no further information on BAV occurrence in this patient is available. Moreover, a single nucleotide substitution in the same codon causing a preterminal stop codon was previously described in a Norwegian patient displaying a classic Marfan phenotype with ectopia lentis, thoracic aorta dilatation and systemic features [15]. According to Polyphen-2 and MuPro, the Arg529Gln mutation is probably damaging and contributes to decreased protein stability. P2 carried two mutations, one of which (Arg2726Trp) has been previously associated with variable clinical phenotypes, including mitral valve prolapse and myopia [14], isolated skeletal features [16], combined skeletal and ocular manifestations [17], mild skeletal abnormalities [18]; and a family in which the mutation appeared incompletely penetrant [19]. The Arg2726Trp mutation was reported in one chromosome in 1000Genomes and NHLBI Exome Variant Server databases as rs61746008 (http://www.1000genomes.org/ and http://evs.gs.washington.edu/EVS/). The other mutation (Arg636Gly) identified in P2 has never been reported in the literature, although another single nucleotide substitution responsible for a different aminoacid change at the same codon (Arg636Ile) in a MFS patient with aortic root dilatation, ectopia lentis and minor involvement of skeleton was previously described [17]. According to SIFT, both the Arg2726Trp and Arg636Gly mutations are classified as damaging, with decreased protein stability as evaluated *in silico* by MuPro.

It is unknown at present if the two mutations at the *FBN1* locus identified in our patient are *in cis*, on the same chromosome, or *in trans*. The infrequent presence of double mutations has been reported in many human gene-causing diseases such as hypertrophic cardiomyopathy [20,21] and arrhythmias associated with the lamin A/C (*LMNA*) gene [22].

The detection of *FBN1* point mutations in patients with BAV with aortic dilatation/aneurysm but without MFS adds to the striking clinical heterogeneity of type I fibrillinopathies to include a small number of patients bearing the most common congenital heart disease. These data also provide further evidence of the heterogeneity of the BAV syndrome [12], with demonstration that aortic dilatation/aneurysm develops in a subgroup of patients as a manifestation of an inherited connective tissue disorder, including *FBN1* mutations in a minority of patients. It is noteworthy that the two patients carrying the mutations displayed a family history of TAA in one and MVP in the other, without systemic features which were otherwise prevalent in the remaining subjects. Moreover, both had aortic aneurysm size attaining the threshold for surgery notwithstanding the young age [23,24], with the largest diameter localized at the level of the sinuses of Valsalva, which is the less prevalent phenotype of aortic dilatation in BAV individuals [1,25]. Della Corte, et al., first named this pattern as “root phenotype” [25] and have subsequently demonstrated that it may be a marker of more severe aortopathy warranting closer surveillance [26]. Interestingly, 8 out of our 10 patients displayed this phenotype, in association with a certain degree of systemic characteristics suggestive of a connective tissue disorder. Finally, the 2 patients bearing FBN1 mutations had significant aortic regurgitation, which is a powerful predictor of loss of aortic medial elastic fibers in patients with ascending aortic aneurysms and aortic valve disease [27]. These findings call for greater focus on the BAV-related cardiovascular abnormalities rather than on the MFS-like systemic features, which may well coexist and warrant investigation in BAV patients in general, but are not associated with the FBN1 mutations identified in the present study. On the other hand, these FBN1 mutations do not completely fulfill the definition of the major criterion for MFS according to the revised Ghent criteria because they have never been detected in Marfan patients with TAA [4]. Therefore our two BAV/TAA patients did not achieve the diagnosis of MFS.

The *FBN1* gene has been previously associated with various conditions, including MFS, neonatal MFS, Shprintzen-Goldberg syndrome, marfanoid neonatal progeroid syndrome, familial arachnodactyly, ectopia lentis, isolated ascending aortic aneurysm and dissection, aortic root dilatation without dissection, skeletal and skin abnormalities (MASS phenotype), Marfan-like syndromes, autosomal dominant Weill-Marchesani syndrome (WMS), mitral valve prolapse, and sclerodermia. Recently, mutations in the *FBN1* gene were reported in two other syndromes [28,29]. The interfamilial clinical heterogeneity at the *FBN1* locus is further characterized by a striking intrafamilial variability (OMIM*134797).

Contrasting data have been reported regarding the genetic background of BAV-related aortopathy. A decrease in *FBN1* mRNA and protein has been demonstrated in some BAV patients suggesting a possible involvement of *FBN1* with BAV [10]. Moreover, single nucleotide polymorphisms (SNPs) spread in the area of the *FBN1* gene, which predispose to TAA, have been reported [30]. On the other hand, other investigators have screened BAV patients for mutations in *FBN1*, *TGFBR2*, and *TGFBR1* genes and failed to detect any mutation, concluding that the *FBN1* gene is not, or only rarely, associated with BAV [31]. More recently, a mutation in the *TGFBR2* gene was reported in a patient classified as aortic dilatation/aneurysm but otherwise not well defined clinically [32]. Another recent study, comparing gene expression in subjects with BAV and tricuspid aortic valves, reported an increase of *FBN1* mRNA only in the subjects with tricuspid aortic valves [33]. Thus, it is conceivable that BAV represents the phenotypic manifestation of many distinct clinical outliers underlined by genetic, molecular, and structural anomalies that do not follow a common path [7].

At present we cannot exclude a coincidence of a common trait such as BAV in males and a rare trait like MFS in our patients. In fact, a limitation of our study is the lack of genomic DNA from parents and other relatives of the two patients carrying mutations in *FBN1* gene to demonstrate their segregation with BAV in the two families. Another limitation of our study is the use of transthoracic echocardiography for the ascertainment of BAV rather than advanced imaging methods. However, the echocardiographic evaluation of our patients was performed by an operator with a wide experience in BAV diagnosis.

Our findings may have relevant clinical implications in the future, if confirmed by larger studies. Although multiple similarities have been shown between MFS and BAV patients, recent improvements in knowledge regarding the natural history of the aortopathy in BAV [34-36] have raised concerns regarding the direct application of surgical criteria adopted in MFS patients to those with BAV and aortic dilatation/aneurysm [37,38]. On the other hand, cardiovascular events are considerable in patients with *FBN1* mutations and remain so throughout life, with men appearing to be at higher risk for an aortic event than women [39]. While we acknowledge that the size of the present study and its retrospective nature do not allow conclusions regarding the indications for surgery, our findings support the need of future studies aimed to characterize BAV patients with aortic dilatation/aneurysm on the basis of their aortic phenotype and other clinical stigmata of connective tissue disorders, eventually performing genetic testing when appropriate. Whether such an approach would result in a different outcome, thus affecting therapeutic choices in patients with BAV and aortic dilatation/aneurysm, should be a pivotal aim of such future research.

## Conclusions

The novel implication of the present findings is the need of a multidisciplinary approach (including internal medicine, medical genetics, cardiology, ophthalmology, cardiovascular surgery, orthopaedic, and molecular biology experts) in the global assessment and management of patients with BAV. Due to the high prevalence of this disorder and its multispecialty requirements, our findings suggest a tailored diagnostic and therapeutic approach, which should be addressed in future prospective studies.

In conclusion, this current findings expand the concept that BAV is a heterogenous disorder with a wide spectrum of clinical manifestations. Beyond the common phenotype of isolated BAV and the previously reported association of BAV in MFS patients [9], we described BAV patients in whom the clinical diagnosis of MFS has been excluded, carrying mutations in *FBN1* gene. These data also expands the clinical spectrum of the type 1 fibrillinopathies to include BAV. These results underscore the importance of accurate clinical characterization of BAV aortopathy, including investigation of systemic connective tissue manifestations and genetic testing.

## Competing interests

The authors declare that they have no competing interest.

## Authors’ contributions

All authors satisfy the requirements for authorship and contributorship. Conception and design: GP, SN, ROB, MY, RA, GFG; Analysis and interpretation BG, ES, MA; Data Collection GP, SN, CP; Writing the article GP, SN, BG, ES; Critical revision of the article GP, SN, ROB, RA, MY, GFG; Final approval of the article GP, SN, BG, ES, MA, CP, ROB, MY, RA, GFG; Obtaining funding RA GFG. All authors read and approved the final manuscript.

## Pre-publication history

The pre-publication history for this paper can be accessed here:

http://www.biomedcentral.com/1471-2350/15/23/prepub
